# Supplementary material for: Recombinant BCG Expressing the Subunit 1 of Pertussis Toxin Induces Innate Immune Memory and Confers Protection against Non-Related Pathogens
Source: Vaccines (Basel). 2022 Feb 3;10(2):234. doi: 10.3390/vaccines10020234 (PMC8879706; doi:10.3390/vaccines10020234)
Supplement: Supplementary file 1 [file vaccines-10-00234-s001.zip › vaccines-1545250-supplementary.pdf]

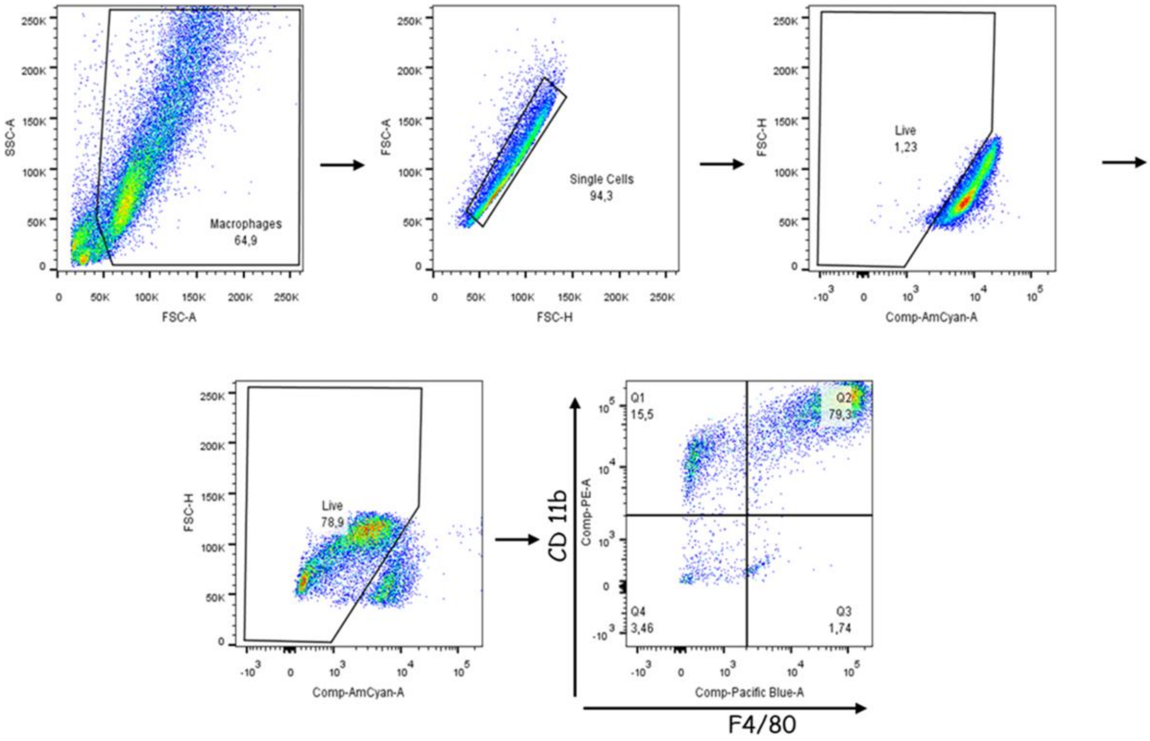

Figure S1: Gating strategy to identify macrophages and their viability. Cell suspensions from the peritoneal cavity and differentiated bone marrow cells were gated for single cells and then live/dead cells according to a negative selection gate based on dead cells (with methanol). Live cells double positive for $CD11b^{+}$ and $F4/80^{+}$ were considered as live macrophages.

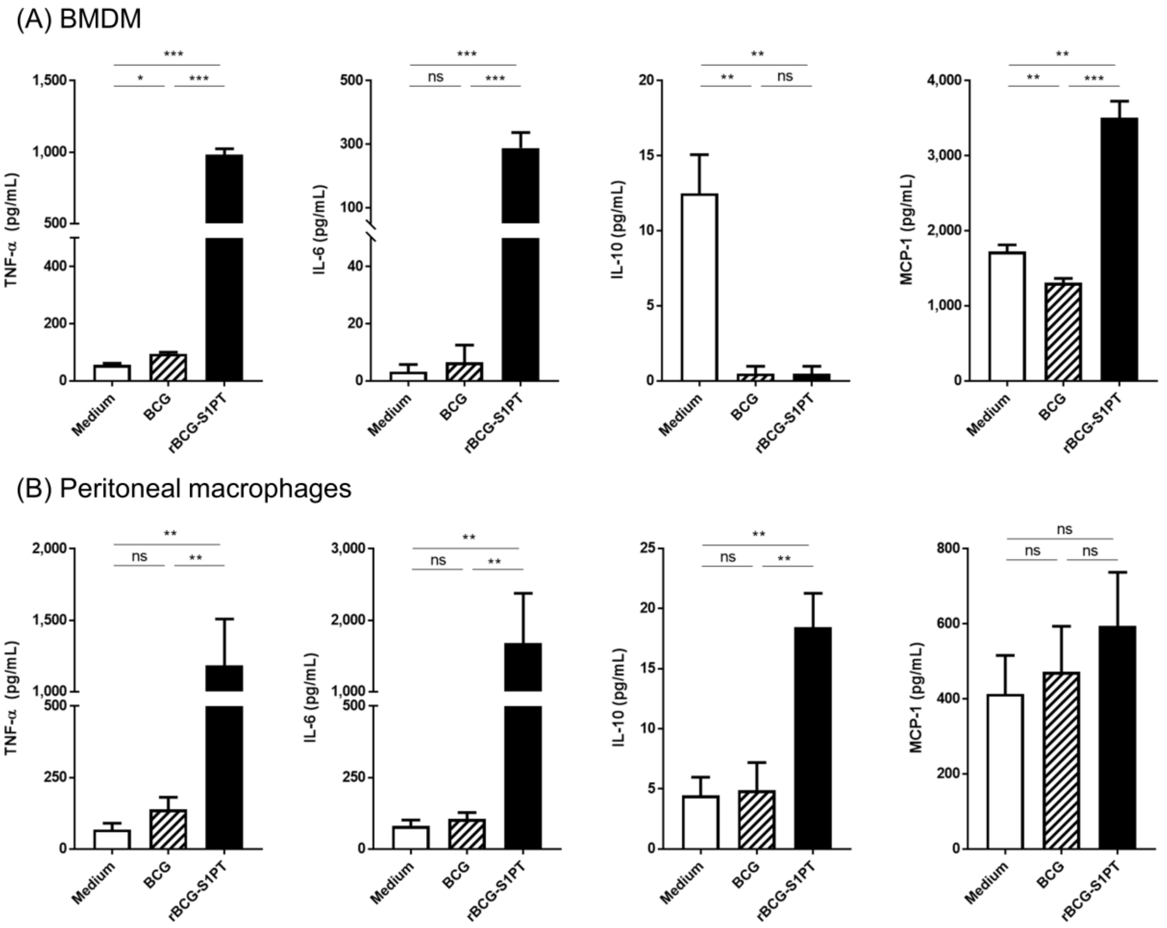

Figure S2: Activation state of primed BMDM and peritoneal macrophages after the 6-day resting phase. Macrophages were exposed for 24 h to culture medium, alone (medium) or containing BCG or rBCG-S1PT at MOI 0.1:1. The cells were then washed and incubated in fresh medium for 6 days, changing the medium on day 4. The supernatants were collected on day 6, and the cytokines produced in the days 5–6 were measured. Statistical analysis was performed via Mann–Whitney U test. * $p < 0.05$, ** $p < 0.01$, *** $p < 0.001$, ns = not significant. Bars represent mean ± SEM of 5–7 replicate samples.

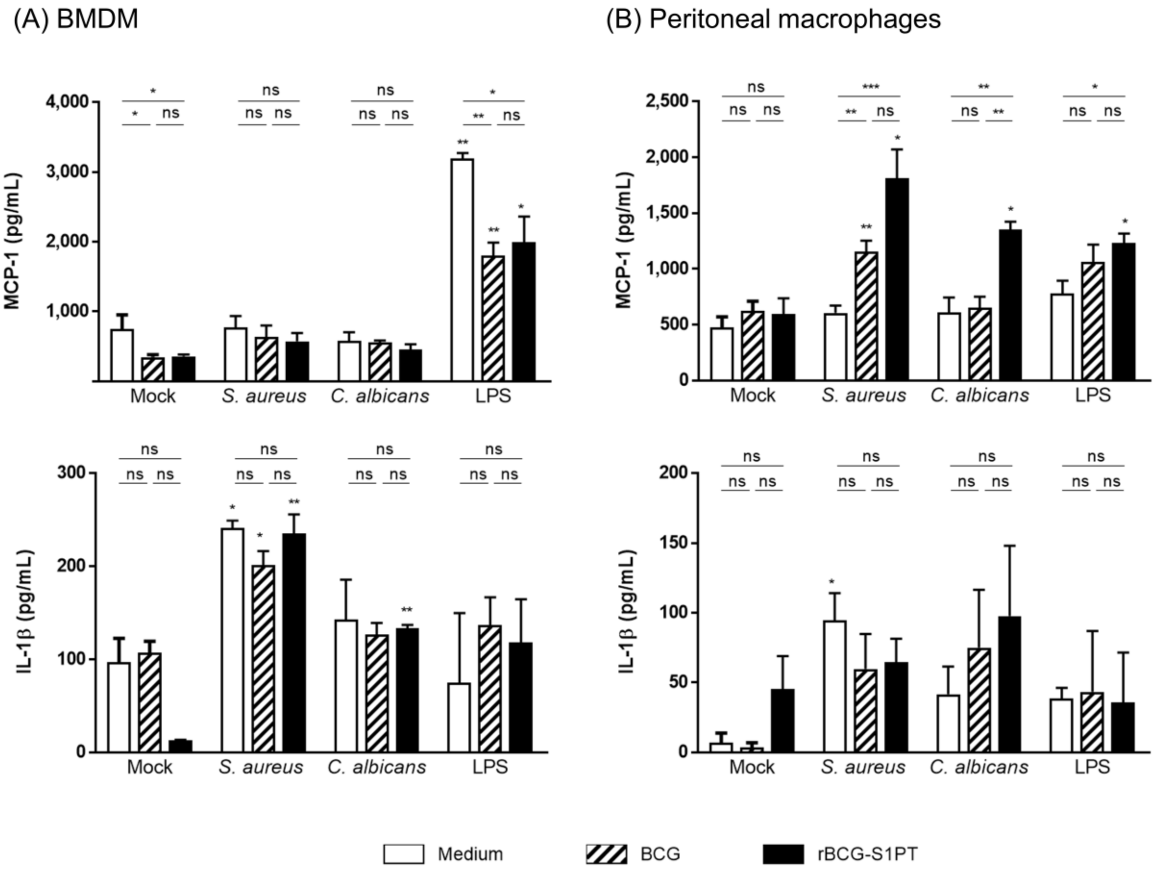

Figure S3: Memory response of primed macrophages to heterologous challenges. BMDM (A) and peritoneal macrophages (B) from naïve mice were exposed in vitro to culture medium alone (unprimed, white bars), BCG (striped bars), or rBCG-S1PT (black bars) (MOI 0.1:1) for 24 h. The cells were then left to rest for 6 days and re-stimulated with medium alone (mock) or with *S. aureus*, *C. albicans*, or LPS (horizontal axis). The production of MCP-1 (upper panels) and IL-1β (lower panels) was measured after 24 h. Statistical analysis was carried out via Mann–Whitney U test. * p <0.05, ** p <0.01, *** p < 0.001, ns = not significant. Asterisks over the columns in *S. aureus*, *C. albicans*, and LPS represent the comparison with the respective mock. Bars represent mean ± SEM of 6–7 replicate samples.

Table S1. Macrophage viability in culture following exposure to BCG and rBCG-S1PT at different MOIs.

(initial 96.1%)		T2 (after BCG stimuli)	T3 (before 2nd stimuli)	T4 (after 2nd stimuli)		
				S. aureus	C. albicans	LPS
BMDM	BCG MOI 0,1	83.4	60.2	58.7	39.9	52.2
	rBCG MOI 0,1	82.8	58.0	70.5	51.1	62.6
	BCG MOI 1	66.5	40.6	53.5	44.4	61.7
	rBCG MOI 1	40.2	26.5	39.9	39.1	39.5
	Cells only	86.3	47.6	46.7	37.3	48.5
PM						
	BCG MOI 0,1	67.9	77.9	48.3	24.2	45.0
	rBCG MOI 0,1	62.8	56.7	51.8	28.6	43.5
	BCG MOI 1	47.5	45.4	26.1	28.5	45.8
	rBCG MOI 1	42.3	34.9	11.9	11.2	11.9
	Cells only	74.8	65.0	57.7	11.6	47.4
